# Pyrolytic Remediation and Ecotoxicity Assessment of Fuel-Oil-Contaminated Soil

**DOI:** 10.3390/toxics10050245

**Published:** 2022-05-12

**Authors:** Byeongwook Choi, Jin-Seo Yu, Gu-Young Kang, Tae-Yong Jeong, Eun Hea Jho, Sung-Jong Lee

**Affiliations:** 1 Department of Environmental Science, Hankuk University of Foreign Studies, 81 Oedae-ro, Mohyeon-eup, Cheoin-gu, Yongin-si 17035, Korea; iop811@naver.com (B.C.); jinsuh.yoo@gmail.com (J.-S.Y.); kanggy@hufs.ac.kr (G.-Y.K.); 2 Department of Agricultural and Biological Chemistry, Chonnam National University, 77 Yongbong-ro, Buk-gu, Gwangju 61186, Korea; ejho001@jnu.ac.kr

**Keywords:** oil-contaminated soil, pyrolysis, TPH, PAHs, Alk-PAHs, UCM, ecotoxicity assessment

## Abstract

Oil-contaminated soil is a major societal problem for humans and the environment. In this study, the pyrolysis method was applied to oil-contaminated soil used as a landfill and gas station site in Korea. The removal efficiency of the main components of oil-contaminated soils, such as total petroleum hydrocarbons (TPH), polyaromatic hydrocarbons (PAHs), unresolved complex mixture (UCM), and alkylated PAHs (Alk-PAHs) were measured, and the effect of temperature, treatment time, and moisture content on pyrolysis efficiency was studied. In order to evaluate the risk of soil from which pollutants were removed through pyrolysis, integrated ecotoxicity was evaluated using *Daphnia magna* and *Allivibrio fischeri*. The chemical and biological measurements in this study include contaminants of emerging concerns (CECs). Results showed that the pyrolysis was more efficient with higher treatment temperatures, moisture content, and treatment times. In addition, toxicity was reduced by 99% after pyrolysis, and the degree of toxicity was evaluated more sensitively in *Allivibrio fischeri* than in *Daphnia magna*. This study shows that weathered oil-contaminated soil can be effectively treated in a relatively short time through pyrolysis, as well as provides information on efficient conditions and the assessment of ecotoxicity.

## 1. Introduction

Petroleum hydrocarbon pollutants create significant environmental impacts in the soil and water environment and pose significant risks to both humans and other forms of life [1]. Petroleum products, including fuel oils, can pose a serious threat to ecosystems and human health, and the remediation of petroleum-contaminated soil may take years, if not decades, and disturb the delicate balance of the existing ecosystem [2]. Total petroleum hydrocarbons (TPH) and polycyclic aromatic hydrocarbons (PAHs) are the main types of contaminants that can be identified in crude-oil-contaminated soil [3]. TPH and PAHs are known to be harmful to human health and the environment [4]. PAHs, in particular, have been found to be toxic and carcinogenic [5].

More importantly, it is reported that alkylated-PAHs (Alk-PAHs), which are generated by the weathering of oil-contaminated soil, are more toxic than PAHs [6]. Research on Alk-PAHs in oil-contaminated soil has received great attention in recent years [7]. Most hydrocarbon compounds contained in crude oil are unidentifiable, and this mixture of unidentified hydrocarbons is called unresolved complex mixture (UCM) [8]. Most organic compounds in UCM have been thought to be harmless, while a few components have shown to pose risks to organisms [9]. Therefore, it is necessary to remove Alk-PAHs and UCM, as well as the main components of TPH and PAHs, when remediating oil-contaminated soil.

Among various remediation techniques, land farming, soil washing, thermal desorption, and phytoremediation are frequently used techniques for oil-contaminated soil, and thermal desorption and pyrolysis can be successfully applied to remove petroleum-derived contaminants within a relatively short treatment time [10,11]. In a previous study, sequential soil washing and biopiling processes took 16 d to remove about 79% of TPH from oil-contaminated soil in Kuwait [12]. In another study, when three bioremediation treatments (landfarming, biostimulation, and biostimulation with bioaugmentation) were used, the removal rates of PAHs over 129 days were 87%, 89%, and 87%, respectively [13]. In comparison, when diesel-contaminated soil was treated by pyrolysis at 250 °C, the TPH concentration was decreased from 6272 mg/kg to 359 mg/kg (i.e., ~95% removal), within 10 min [14]. With a thermal treatment such as pyrolysis, higher contaminant removal efficiency can be achieved in a shorter treatment time. Pyrolysis operates at lower temperatures and anoxic atmospheric conditions compared to conventional thermal treatment methods, during which large organic molecules can be broken down into smaller molecules that can be more easily removed [15]. In addition, pyrolysis can be applied to remove a wide range of pollutants and leaves the soil intact for future use, and for these merits, pyrolysis is receiving much attention as an important soil remediation method [16].

Many studies are conducting pyrolysis studies by artificially mixing crude oil into the soil [17,18,19]. Contaminated sites have different soil properties, moisture content, and pollutant concentrations from a lab environment; however, studies applying pyrolysis to the field samples have been limited. In addition, validation of the contaminant removal efficiency in terms of toxicity may be required in order to reuse the treated soil because some unexpected residual compounds can impose toxic effects. The purpose of this study is to find the optimal condition by evaluating the efficiency by moisture content, temperature, and operating time to apply the high-efficiency but energy-consuming pyrolysis method among oil-contaminated soil remediation technologies. Therefore, this study investigated application of pyrolysis to a fuel-oil-contaminated field sample and evaluated the pyrolysis efficiency under different soil moisture content, the pyrolysis temperature, and the operational time.

The soil sample used in the study was collected from a site that was once used as a landfill from the 1960s to the 1970s, and then a gas station from 1988 to 2010. We measured both contaminants of emerging concern (CECs) and conventional contaminants. PAHs including benzo(e)pyrene were measured using a targeted approach. Non-targeted approaches were also applied to cover unknown contaminants or known CECs in analyses of the soil contaminant mixture (TPH and UCM). The analyses were applied to the soil samples before and after the pyrolysis treatment. At the same time, the toxicity of untreated and treated soils was determined using two indicator organisms, *Daphnia magna* and *Allivibrio fischeri*. By measuring toxicity, the presence of any undegraded contaminants including CECs was measured that were not covered by chemical analyses.

## 2. Materials and Methods

### 2.1. Characteristics of the Soil Sample

To collect a wide range of contaminated sample at a depth of 5–10 m, the surface soil was sampled using a mechanical device and the contaminated soil was well mixed and collected. Once collecting a part of mixed sample, the sample was placed in a dark room under dry atmospheric conditions for one day, and then non-soil material and large soil particles were separated using a 2 mm sieve. The pressure plate method (PPA), a standard technique among many methods for estimating the water holding capacity (WHC) of the soil, was used [20]. For the soil texture, the commonly used hydrometer method was used [21]. The water holding capacity (WHC) of the soil was 43.2% and the pH was 6.99 ± 0.02. The soil was composed of 66.4% sand, 19.8% clay, and 13.7% silt (i.e., sandy clay loam). The initial concentrations of TPH, UCM, PAH, and Alk-PAHs were 2092 ± 19 mg/kg, 1488 ± 19 mg/kg, 0.89 ± 0.17 mg/kg, and 71 ± 6.78 mg/kg, respectively.

### 2.2. Pyrolysis Experiment

**Figure 1:**
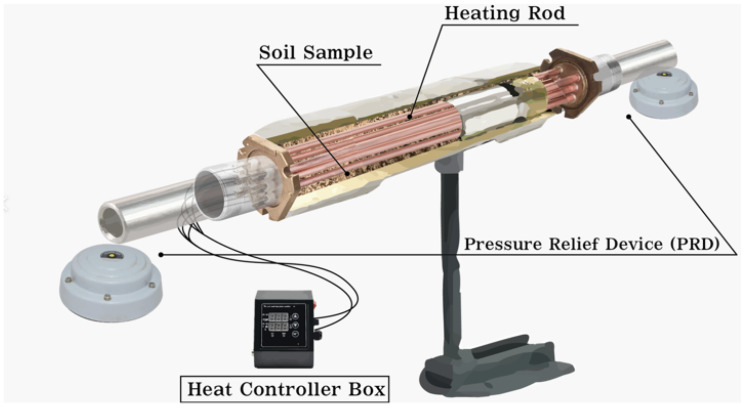
Example of pyrolysis reactor used in this study.

Figure 1 shows the pyrolysis device used in this study. The length of the pyrolysis device is 25 cm and the diameter is 3.5 cm, and it is made of stainless steel (SUS304). The indirect heating method was used, which controls the temperature of the heating rods in the control box, allowing indirect heating of the soil sample.

As a result of measuring the moisture content of soil containing natural moisture, it was found to be around 10–20%. Before proceeding with the pyrolysis experiment, the soil sample was divided into two groups, one with a moisture content of 10% and the other with a moisture content of 20%. The soil sample (10 g) was then placed in the pyrolysis device and the soil was pyrolyzed under different temperatures (200, 400, 600 °C) for different operational times (30, 60 min), resulting in a total of twelve moisture-content–temperature–operational time conditions. After pyrolysis, the soil samples were removed from the pyrolysis device and then placed in desiccators to cool down. Each experiment was repeated three times, and the average value of the measured concentrations were used to calculate the removal efficiency (*R_e_*) using Equation (1).

(1) $$R_{e}\left(\%\right)=\frac{\left(C_{0}-C\right)}{C_{0}}\;\times 100$$

where *C*_0_: the initial pollutant concentration of the soil (mg/kg) and *C*: the pollutant concentration of the soil (mg/kg) after pyrolysis. Statistical evaluation was performed by Independent Sample *t*-test using the software SPSS statistics 17.0.

### 2.3. Analytical Methods

Extraction and purification of the soil samples were carried out according to the quantitative oil analysis method [22]. TPH, UCM, PAHs, and Alk-PAHs were extracted with n-hexane (95%, J.T. Baker) using a Soxhlet apparatus for 16 h. The residual TPH and UCM concentrations in the extracts were analyzed using a gas chromatograph equipped with a flame ionization detector (GC-FID; Agilent 6890 N, Agilent, Delaware USA). For TPH analysis, the surrogate standard of o-terphenyl and the internal standard of 5α-androstane were used. The residual concentrations of PAHs and Alk-PAHs in the extracts were analyzed using GC equipped with a mass spectrometry (GC–MS; Agilent 6890/HP 5973, Agilent, Delaware, USA). For the analysis of PAHs and Alk-PAHs, dibenzothiophene-D8 and PAHs internal standard 5 mix (naphthalene-D8, acenaphthene-D10, phenanthrene-D10, chrysene-D12, perylene-D12) were used as the surrogate standards, and *p*-terphenyl-D14 was used as the internal standard. All standards were purchased from Accustandard. The analytical conditions of GC-FID and GC-MS are shown in Table 1.

### 2.4. Bioassay

In this study, ecotoxicity evaluation was performed using *Daphnia magna* and *Allivibrio fischeri*. Toxicity was evaluated by comparing the change in toxicity before and after contaminant treatment on oil-contaminated soil. In the oil-contaminated soil used in this study, oil had very low solubility in water. In order to conduct ecotoxicity evaluation, considering the physical/chemical characteristics of fuel oil, the following three toxicity test methods were applied: whole soil (solid-phase), organic extraction, and aqueous extraction. The water extracts were used to test the mobile fraction of the contaminants, while the soil slurry was used to test the bioavailable fraction of the contaminants. The organic extracts are used to represent the total organic toxicity of the contaminated soil. Whole soil samples were prepared by mixing contaminated soil and deionized water (solid-to-liquid ratio of 1:4) for 30 min and settling in a refrigerator at 4 °C for 24 h. The water extracts were prepared by mixing the contaminated soil and deionized water at 1:5 (*w*/*v*) solid-to-liquid ratio for 24 h followed by centrifugation to sample the supernatant. Organic soil extracts were prepared by an 8 h Soxhlet extraction with hexane and acetone mixture (1:1 (*v*:*v*)). The extracts were resuspended in dimethyl sulfoxide (DMSO). The highest DMSO concentration used in the bioassay was 1% (*v*/*v*) [23]. Concentrations of 1% were not toxic to *Daphnia magna* and *Aliivibrio fischeri* [24]. All toxicity assessments were performed in accordance with US EPA whole effluent toxicity (WET) guidelines.

#### 2.4.1. Toxicity Tests Using *Daphnia magna*

The *Daphnia magna* used in the experiment is an organism cultured in a laboratory maintained at 20 °C, and quality control is maintained using potassium dichromate. The culture medium for *Daphnia magna* contained KCl (8 mg/L), CaSO_4_ 4H_2_O (120 mg/L), MgSO_4_ (120 mg/L), and NaHCO_3_ (192 mg/L) to maintain the hardness at 160–180 mg/L as CaCO_3_ and the pH value at about 7.6–8.0. Toxicity tests were conducted by a serial dilution of the samples with the culture medium. The sample dilution rates (% *v*/*v*) used in the toxicity tests were 100%, 50%, 25%, 12.5%, 6.25%, and 0% (i.e., negative control, 100% of culture medium). EC50 was calculated using toxcalc version 5.0 software(Tidepool scientific software, McKineyville, USA), and the calculated EC50 value was converted into Toxic Units (TU) to represent the toxicity value (TU; 100/EC50).

#### 2.4.2. Toxicity Tests Using *Allivibrio fischeri*

Toxicity tests were carried out using *Allivibrio fischeri* according to the ISO 11348–3:2007 method. The changes in the bioluminescence before and after exposure to the sample were measured using the Microtox M500 (Modern Water INC, Cambridge, UK). *Allivibrio fischeri* were evaluated for 5 min acute toxicity by applying the 81.9% method. Samples were measured for bioluminescence for 9 serially diluted samples from 81.9%. The half maximal effective concentration (EC50) for each condition was determined using the Microtox Omni software (Modern Water INC, Cambridge, UK), and the calculated EC50 value was converted into TU to represent the toxicity value. The luminescence inhibition rate was calculated as follows in Equation (2).

(2) $$\mathrm{Inhibition}\;\left(\%\right)=\left(1-\frac{I_{t}}{I_{0\;}\times R_{t}}\right)$$

where *R_t_*: the correction factor obtained when the bioluminescence intensity of the control after time *t* is divided by the initial intensity of the control. *I_t_*: The bioluminescence intensity of the samples after time *t*. *I*_0_: The initial bioluminescence intensity of the samples.

## 3. Results and Discussion

### 3.1. Removal Efficiency of TPH and UCM

**Figure 2:**
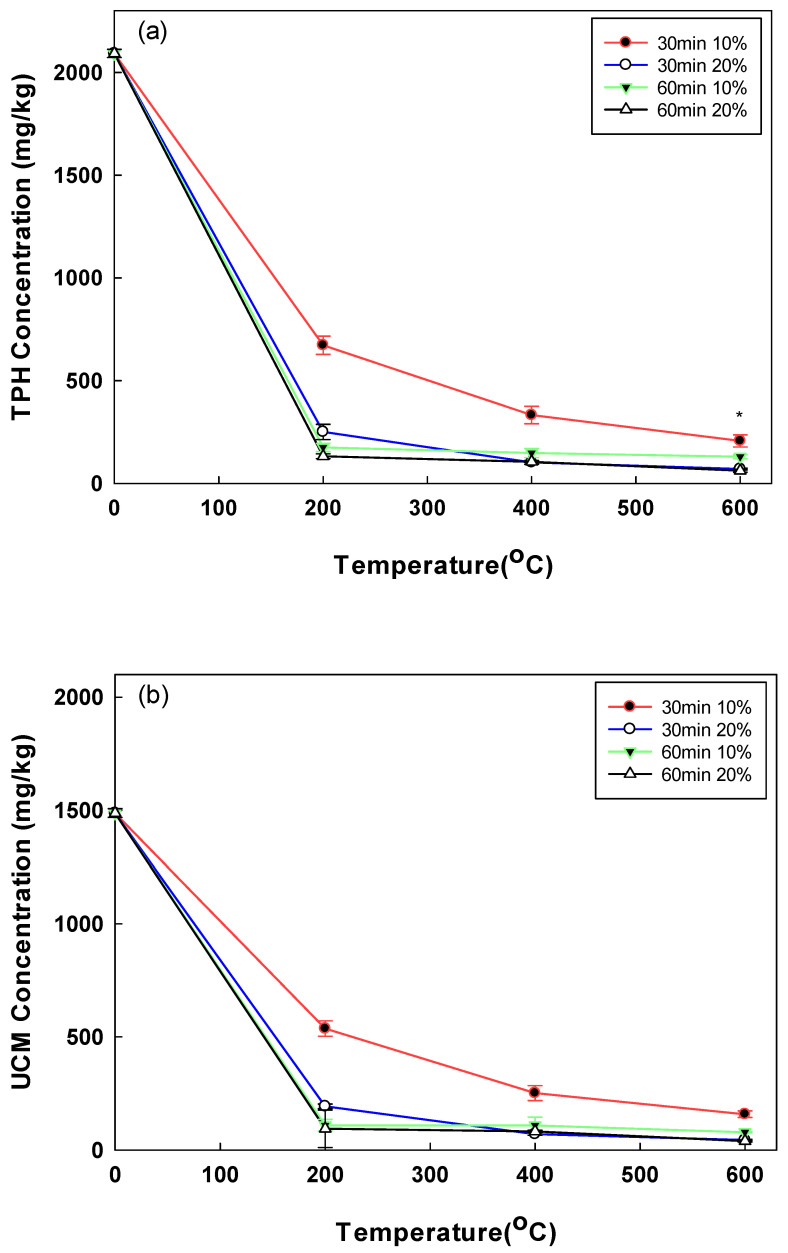
(**a**) Post-pyrolysis TPH concentration by operational time, temperature, and soil moisture content; (**b**) post-pyrolysis UCM concentration by operational time, temperature, and soil moisture content (* *p*-value < 0.05).

TPH and UCM concentrations represent the contamination by mixtures of CECs and conventional oil contaminants. The changes in the concentrations of TPH and UCM at different soil moisture contents and pyrolysis temperature conditions are shown in Figure 2. For the 30 min pyrolysis, the TPH removal increased with the temperature rising from 200 °C (68% at moisture content of 10%) to 600 °C (90% at moisture content of 10%). The UCM showed a similar trend. In addition, the TPH and UCM removals were higher in conditions with higher moisture content. For example, at 400 °C, the TPH removal increased from 85% at the moisture content of 10% to 95% at the moisture content of 20% (Figure 2a). The maximum TPH and UCM removal rates were obtained when the soil samples with the moisture content of 20% were pyrolyzed at 600 °C for 60 min. As a result of pyrolysis at 600 °C for 60 min, the residual amount of TPH was not statistically significant as a result of the t-test, according to the difference in moisture content (*p*-value = 0.001). In our previous study on the open system pyrolysis, the TPH removal rates of 74% and 76% were obtained after 30 min and 60 min treatments at 200 °C [25]. In this study, the closed system pyrolysis achieved higher TPH removal rates (i.e., 88% and 93% after 30 min and 60 min, respectively). Another previous study reported 70% TPH removal from an oil-contaminated soil sample after treatment at 300 °C for 10 min [26]. In contrast to a previous study, as a result of controlling the moisture content in the pyrolysis method in the case of TPH when it was operated at 200 °C for 60 min, it was possible to confirm the high removal efficiency of 91.61% and 93.68% at the water content of 10% and 20%, respectively [25]. In addition, the residual amounts of TPH and UCM decreased significantly up to 200 °C, but it was confirmed that sharpness decreased when applied at a higher temperature. This can be used as an indicator of minimal cost, requiring high energy costs in the pyrolysis method.

It is known that the UCM, which is commonly used as an indicator of petroleum pollution, contains more than 250,000 compounds [21]. UCM contributed to the overall toxic effect of oil on marine amphibians [27,28]. Additionally, recent studies have indicated that UCM compounds may have adverse effects on aquatic organisms and are environmentally hazardous [29]. Research on UCM with complex composition and many compounds is still lacking. Therefore, it is important to remove UCM, as well as TPH and PAHs, when applying oil-contaminated soil remediation technology.

**Figure 3:**
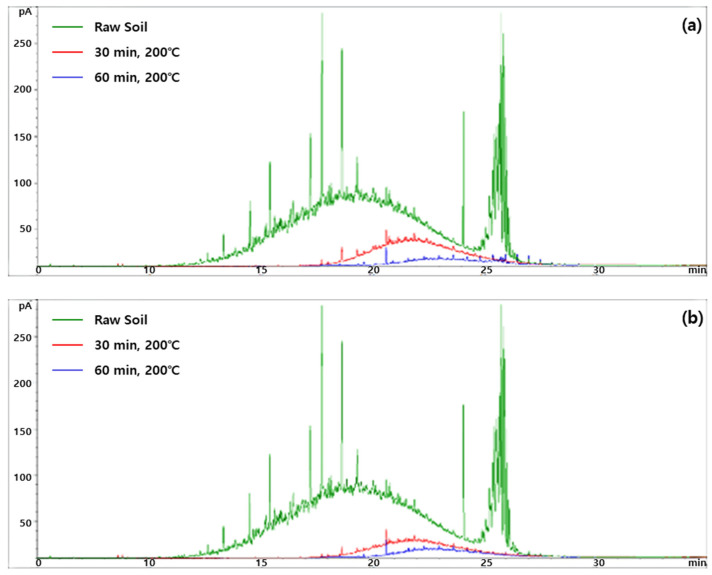
GC chromatograms of the TPH and UCM. (**a**) Moisture content 10%, temperature 200 °C; (**b**) moisture content 20%, temperature 200 °C. The green, red, and blue lines indicate the chromatograms of the raw soil, 30 min treated soil, and 60 min treated soil, respectively. The area under the hump is considered as the UCM.

In previous studies, UCM was shown as humps in GC chromatograms, and only the GC-resolved peaks in GC chromatograms were used to determine the total detectable TPH [30]. GC chromatograms of the TPH and UCM are shown in Figure 3. The large hump of the raw soil (untreated soil) chromatogram indicates a large amount of UCM, and this was significantly reduced after heat treatment at 200 °C for 30 and 60 min (Figure 3a). The humps decreased further at higher moisture content (i.e., 20%) (Figure 3b). Overall, the removal efficiencies of TPH and UCM were higher at the moisture content of 20% than at 10%. Water molecules are highly polar and easily occupy the adsorption site of the soil, so many contaminants can be removed from the soil, and the water will increase rapidly while heating the soil, making it more efficient to remove contaminants from the soil [31]. When the moisture content of the soil is more than 20%, water evaporation can increase heat loss during the heating process, which can increase the treatment cost [32].

### 3.2. Removal Efficiency of PAHs and Alk-PAHs

**Figure 4:**
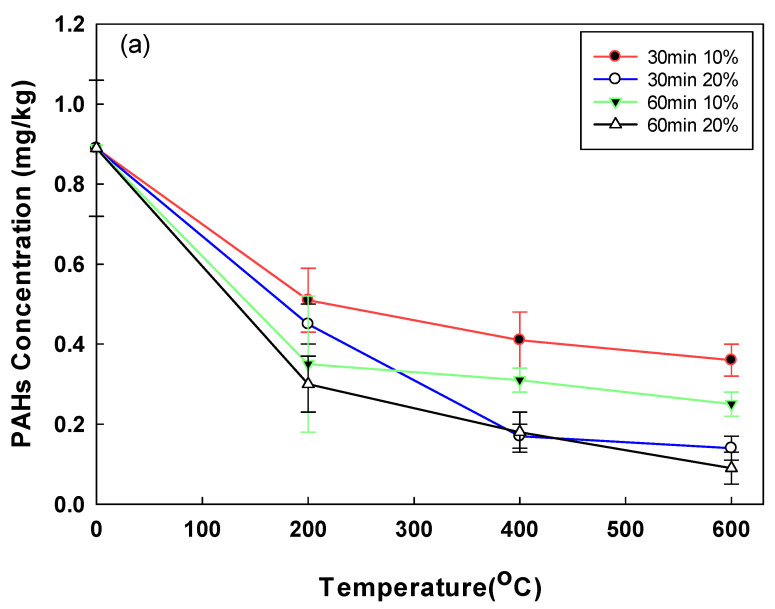

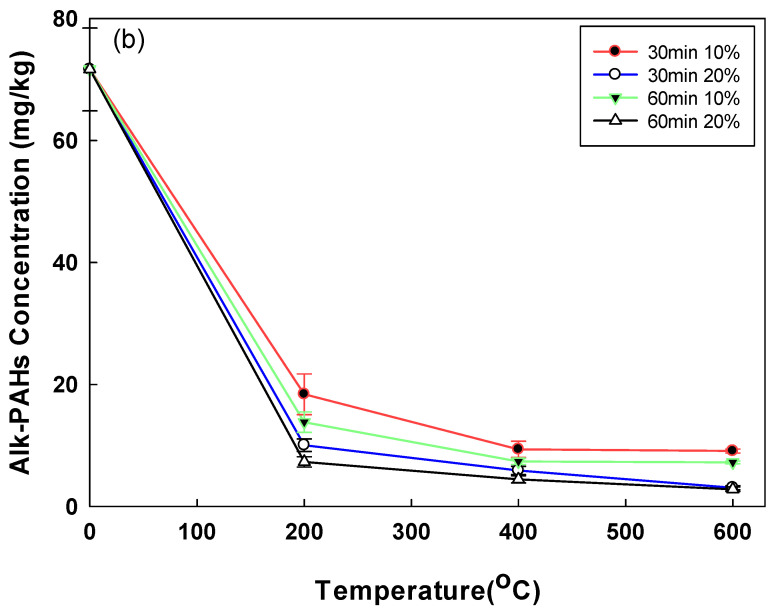
(**a**) Post-pyrolysis PAHs’ concentration by operational time, temperature, and soil moisture content; (**b**) post-pyrolysis Alk-PAHs’ concentration by operational time, temperature, and soil moisture content.

**Figure 5:**
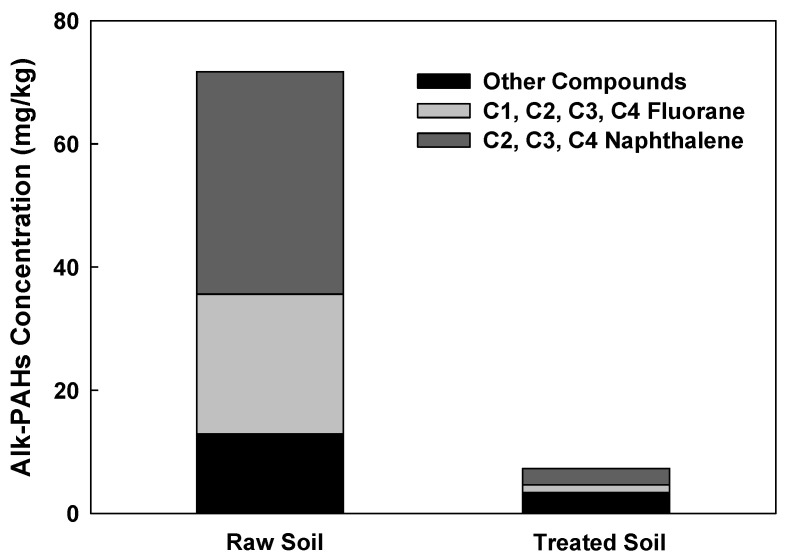
Content of fluorane, naphthalene, and other compounds in raw soil and treated soil.

The removal of PAHs and Alk-PAHs in the soil samples is shown in Figure 4. A total of 17 PAHs including a CEC, benzo(e)pyrene, were analyzed. The initial concentration of PAHs in the soil was comparatively lower than the concentrations of the other pollutants (TPH, UCM), which could probably be due to the weathering of PAHs [33]. PAHs, which had an initial low concentration of 0.89 mg/kg due to soil weathering, had a removal efficiency of 42.35% and 66.48%, respectively, when the moisture content was 10% and 20% at 200 °C. PAHs with the highest removal efficiency of 90.31% were obtained when the moisture content, temperature, and operating time were 20%, 600 °C, and 60 min, respectively. The highest removal rate of the Alk-PAHs (i.e., 96%) was observed after a 60 min treatment at 20% moisture content at 600 °C. When the operational time was 30 min at 600 °C, the soil samples of 10% and 20% moisture contents demonstrated a removal efficiency of 59.1% and 84.3%, respectively. For Alk-PAHs, when treated for 30 min at 200 °C, the soil sample with a moisture content of 10% had a removal efficiency of 74.4%, resulting in a concentration of 18.38 mg/kg and 20% moisture content, a removal efficiency of 86%, and a concentration of 10.38 mg/kg. The removal efficiencies of PAHs and Alk-PAHs were higher in conditions with higher moisture content. In the case of oil-contaminated soil for a long time rather than temporary pollution, oil components are strongly attached to the soil. In the pyrolysis method, when a certain amount of moisture is present, the pressure increases as the moisture turns into steam. It is assumed that the oil contaminants strongly attached to soil particles better desorbed when the vapor pressure of moisture content increased. The detached contaminants are vulnerable degradation by heating, and this explains why moisture content affects pyrolysis efficiency. In a study with the change in moisture content for pyrolysis application in solid waste, in order to obtain the best efficiency, it was more efficient to adjust to 20–25% than when the moisture content was 9% in solid waste [34]. In addition, in a study applying the pyrolysis of sewage sludge with different moisture content, it was confirmed that the removal efficiency increased, and toxicity decreased as the moisture content increased [35]. In a previous study, a higher removal efficiency of PAHs was demonstrated when high temperatures were applied, and a similar trend was observed in this study. For example, when PAHs were thermally incinerated at 870–1200 °C, the removal efficiency was greater than 90%, and the PAHs’ removal efficiency of 99.9% was obtained when heated at >450 °C for 1 h using an indirect heating thermal desorption device [36]. Similarly, in this study, the removal efficiency was 66.48% at 200 °C, 20% moisture content, and 60 min operation time, but at 600 °C, under the same conditions, the removal efficiency increased to 90.31%. Among the 29 compounds of Alk-PAHs in raw soil, C2, C3, and C4-naphthalene, and C1, C2, C3, and C4-fluorane accounted for 50% and 32%, respectively. In the treated soil (60 min, 600 °C, 20% pyrolysis), it was confirmed that naphthalene and fluorane accounted for 21% and 12%, respectively, and significantly decreased (Figure 5). The removal efficiency of naphthalene and fluorane, which accounted for a high proportion, was confirmed to be 95–99% removed from the treated soil. Currently, there is insufficient information on the physicochemical and biological treatment processes to remove Alk-PAHs, as well as on how such treatment can affect toxicity of Alk-PAHs [37].

This study evaluated removal efficiency of oil-contaminated soil using pyrolysis. TPH, UCM, PAHs, and Alk-PAHs all showed the highest efficiency when the temperature was 600 °C, moisture content was 20%, and operation time was 60 min. The optimum conditions for the pyrolysis performed in this study were evaluated as 20% moisture content, 600 °C, and 60 min treatment time. Among oil components, TPH, PAHs, and Alk-PAHs showed 97.04%, 90.31%, and 96.05% removal efficiency, respectively. Among the measured PAHs, benzo(e)pyrene is a known CEC [38]. Information on benzo(e)pyrene is currently lacking; therefore, more studies on this compound seem necessary. Additionally, unknown oil contaminants that were measured by TPH and UCM concentrations also require further studies, as this study showed in Figure 3 that unknown contaminants are abundant and a target for remediation in the contaminated soil [30].

### 3.3. Daphnia magna and Allivibrio fischeri Ecotoxicity Assessment

The treated soil was prepared by performing a pyrolysis process with a moisture content of 20%, a temperature of 600 °C, and a treatment time of 60 min. The EC50 values of *Daphnia magna* and *Allivibrio fischeri* were evaluated to be 0.29% and 0.21% before contaminants were removed through pyrolysis, and the EC50 values after contaminant treatment were 76.92% and 37.04%. The final result was expressed by converting the EC50 value into TU (Table 2). The organic phase of extracts from the contaminated soil were strongly toxic for both *Daphnia magna* and *Allivibrio fischeri*. The toxic effects of the whole soil and soil solution were not observed (i.e., TU values of 0), while the soil organic extracts exhibited toxic effects (Table 2). This could be attributed to the high Kow values of oil components. Based on the TU values of soil organic extracts, *Allivibrio fischeri* showed higher sensitivity than *Daphnia magna*. The treated soil had a >99% reduction in toxic effects, both with the *Daphnia magna* and *Allivibrio fischeri*. The decrease in soil toxicity is related to the decrease in pollutants after a treatment at 600 °C. In a study comparing the ecotoxicity of soil artificially contaminated with diesel with an initial TPH of 45,000 mg/L after bioremediation, it was confirmed that the ecotoxicity of *Daphnia magna* before and after treatment was reduced by 300% [39]. Vignet et al. [40] showed that Alk-PAHs had effects on aquatic organisms such as growth inhibition, behavioral disturbances, malformations, and reduced survival rates. Toxicity tests conducted by Turcotte et al. [41] showed that the alkylated form of phenanthrene is more toxic than the parent form, with toxicity increasing with increasing alkyl substituents. In this study, it was confirmed that the toxic effect after pyrolysis in oil-contaminated soil was reduced by >99%, so that it did not affect organisms when the soil was reused after treatment.

## 4. Conclusions

In this study, the removal efficiency of TPH, UCM, PAHs, and Alk-PAHs was confirmed by controlling the treatment time, temperature, and moisture content after fabricating a pyrolysis device with the indirect heating method in the treatment of oil-contaminated soil. Higher temperatures, longer reaction times, and higher moisture content were found to be effective in removing contaminants, with temperature being more effective than reaction time. Through pyrolysis treatment, the maximum removal efficiency of TPH, UCM, PAHs, and Alk-PAHs of 97%, 97%, 90%, and 96% was confirmed at a moisture content of 20% and 600 °C, after the process, respectively. In addition, *Daphnia magna* and *Allivibrio fischeri* toxicity evaluation was performed on the soil before and after the removal of pollutants under the conditions of 20% moisture content, 600 °C, and 60 min treatment time, which showed the highest removal efficiency of oil pollutants; *Daphnia magna* showed a high toxicity removal efficiency of 99.6%, while *Allivibrio fischeri* showed 99.4%. Although the removal efficiency of each compound is different after the pyrolysis process, it was confirmed that the toxicity was significantly reduced as a result of the integrated toxicity evaluation. It is thought that a follow-up study using the toxicity identification evaluation (TIE) method recommended by the US EPA to evaluate the toxicity of individual compounds is necessary.

## Figures and Tables

**Table 1:** Operation conditions of GC-FID and GC-MS.

Conditions	GC-FID	GC-MS
Injector temp.	250 °C	260 °C
Detector temp.	320 °C	300 °C
Flow gas	N_2_	He
Flow rate	1 mL/min	1 mL/min
Injection volume	1 µL	1 µL
Spilt Mode	1:10	-
Columns	Silica capillary column DB-5 (Agilent J&W, 30 m × 0.25 mm id × 0.25 µm, Agilent, Santa Clara, USA)	Silica capillary column HP-5MS Ultra Inert (30 m × 0.25 mm id × 0.25 µm, Agilent, Santa Clara, USA)

**Table 2:** Toxic effects of oil-contaminated soil on *Allivibrio fischeri* and *Daphnia magna*.

	Whole Soil		Soil Organic Extract		Soil Aqueous Extract	
	*Allivibrio fischeri*	*Daphnia magna*	*Allivibrio fischeri*	*Daphnia magna*	*Allivibrio fischeri*	*Daphnia magna*
	TU	TU	TU	TU	TU	TU
**Raw soil**	0	0	475.5 (25.8)	350.7 (23.5)	0	0
**Treated soil**	0	0	2.7 (0.15)	1.3 (0.07)	0	0

## Data Availability

The data presented in this study are available on request from the corresponding author.
